# The mental health age gradient by gender identity

**DOI:** 10.1007/s00127-025-02895-3

**Published:** 2025-04-05

**Authors:** Samuel Mann, Megan S. Schuler, Annaliese Paulson, Michael S. Dunbar

**Affiliations:** 1 RAND, 1200 S Hayes St, VA 22202 Arlington, USA; 2 University of Michigan, Ann Arbor, MI USA; 3 RAND, 4570 Fifth Ave, Suite 600, PA 15224 Pittsburgh, USA

**Keywords:** Gender minority, Transgender, Mental health, Age

## Abstract

Using data from the Behavioral Risk Factor Surveillance System (BRFSS) 2017–2022 we estimated adjusted and unadjusted differences in self-reported number of poor mental health days (past month) between gender minority (GM) and cisgender adults. We document that the disparity is largest among younger individuals. Among 18- to 23-year-olds, GM adults report on average 14.5 days of poor mental health, compared to 6.3 days for cisgender individuals, yielding an unadjusted disparity of 8.2 days [95% CI: 7.15, 9.24]. This disparity decreases with age – among people over the age of 73, the observed difference was 1.7 days [95% CI: 0.13, 3.27].

**Supplementary Information:**

The online version contains supplementary material available at 10.1007/s00127-025-02895-3.

## Introduction

Studies have consistently documented that gender minority (GM) individuals (including transgender, gender diverse, gender non-conforming and gender non-binary) experience worse mental health outcomes than their cisgender counterparts [1]. The minority stress model posits that these disparities are driven in part by increased exposure to internal and external (including both interpersonal and structural) stressors uniquely experienced by socially marginalized individuals [1–3]. Prior studies have documented mental health disparities among GM individuals for specific age groups (e.g., adolescents and young adults (13–24) [4], older adults (> 50) [5]) and for the general adult population [6]. However, little is known regarding how GM mental health disparities vary across the life course. Such research is critical for identifying age periods when targeted interventions may be most needed to reduce disparities and support mental health and well-being for GM individuals.

## Methods

This study used yearly cross-sectional probability-based samples from the Behavioral Risk Factor Surveillance System (BRFSS) 2017–2022 from 43 states implementing the optional sexual orientation and gender identity module. The exposure variable was GM identity, which was elicited from the question ‘‘do you consider yourself to be transgender?’’ Our primary outcome, number of poor mental health days in the past month (0–30), was assessed with the item: “Now thinking about your mental health, which includes stress, depression, and problems with emotions, for how many days during the past 30 days was your mental health not good?”

Age-specific mean number of poor mental health days for both GM and cisgender adults are presented graphically. Statistical testing of disparities was conducted using ordinary least square regression models that were stratified by age (i.e., twelve 5-year age bins). Unadjusted and adjusted models were estimated; adjusted models controlled for whether the respondent was contacted via cellphone or landline, state of residence, survey year, binary gender (assessed with question “are you male or female?”), race (White, Black, Asian, American Indian/Alaskan Native (AIAN), other race), Hispanic ethnicity, education level (no high school diploma, high school diploma, some college, college degree), marital status (married vs. not married), parenthood (yes vs. no), and sexual minority status (identify as lesbian, gay, or bisexual (LGB) vs. heterosexual). All estimates were generated using the BRFSS sampling weights [7]. Statistical analysis was conducted via Stata version 17.0.

The study followed the Strengthening the Reporting of Observational Studies in Epidemiology (STROBE) reporting guidelines and did not require ethical approval from an institutional review board as it used de-identified publicly available data.

## Results

Our pooled analytic sample included 1,378,900 adults of whom 0.5% (*n* = 6,498) were GM adults. Compared with cisgender adults, GM adults were more likely to be younger, were more racially and ethnically diverse, more likely to identify as LGB, had lower levels of education, and were less likely to be married or be a parent (Supplementary Table 1). In aggregate, GM individuals reported a higher number of poor mental health days (9.02 days) compared to cisgender individuals (3.94 days).

**Fig. 1:**
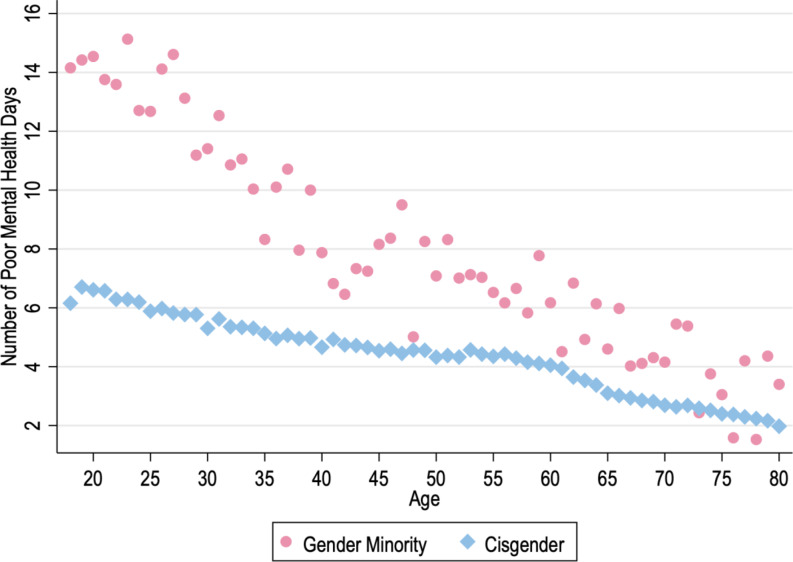
Data come from the Behavioral Risk Factor Surveillance System (2017–2022). Pink circles denote the unadjusted age-specific mean number of poor mental health days among GM’s. Blue diamonds denote the unadjusted age-specific mean number of poor mental health days among cisgender individuals

Figure 1 presents the average number of poor mental health days for GM and cisgender adults across ages 18 to 80. Unadjusted and adjusted models examining disparities across age categories (i.e., 5-year age bins) are presented in Supplementary Tables 2 and 3, correspondingly. Across both unadjusted and adjusted models, the magnitude of the GM mental health disparity is larger among younger adults compared to older adults. Unadjusted estimates indicate an average of 14.5 days of poor mental health for GM adults ages 18–23 compared to 6.31 days for cisgender peers, yielding a disparity of 8.19 days [95% CI: 7.15, 9.23]. Similarly, GM adults ages 23–27 averaged 14.42 days of poor mental health compared to 5.88 days for cisgender peers, reflecting a disparity of 8.54 days [95% CI: 7.18, 9.90]. The observed disparity consistently trends downwards across age – at age 73+, we observe a 1.70 day [95% CI: 0.13, 3.28] disparity.

The observed disparity between GM and cisgender adults is attenuated in adjusted models, ranging from 4.22 days [95% CI: 3.24, 5.19] among adults ages 18–23 to 1.44 days [95% CI: -0.24, 3.12] among adults ages 73+ (Supplementary Table 3). Irrespective of adjustment, the same broad pattern remains – i.e., that the mental health disparity is substantially larger among younger rather than older cohorts.

## Discussion

In line with prior studies and the minority stress model, this study documents that GMs experience significant mental health disparities [1, 2] and further demonstrates that the size of the GM mental health disparity varies substantially across the age gradient. Notably, GM young adults ages 18–23 and ages 23–27 had greater than 14 days of poor mental health days in the past month, which meets criteria for the Centers for Disease Control and Prevention’s (CDC’s) definition of frequent mental distress (14 + days/month). Frequent mental distress can lead to significant impairments in physical health, social functioning, and other aspects of life [8]. Concerningly, the magnitude of the mental health disparity among GM adults was greater than twice that of cisgender peers for ages 18–23, 23–27, and 28–32, indicating that profound disparities persist across young adulthood into their 30s for GM individuals. These findings underscore an urgent need for targeted interventions to address mental health concerns for GM individuals as they navigate early adulthood.

Controlling for observable characteristics reduced the magnitude of observed disparities, implying that at least a portion of the disparity is accounted for by other identity and social factors (e.g. variation across gender identity in educational attainment, race, and sexual identity, all of which are associated with mental health). Such findings highlight the important role of social determinants of health to the mental health disparity experienced by GMs and the potential role of intersectionality in explaining this variation.

Findings also suggest that disparities diminish over the course of adult life. This pattern may be attributable to changes in internal and external stressors faced by GMs over the life course as well as shifts in protective factors (e.g., identity-related resiliency, social support, coping strategies, mental health service use) that contribute to improved mental health over time. In the context of a sparse literature on GM well-being across developmental periods, additional research that takes a life-course perspective to understanding factors that contribute to GM health and well-being is needed to inform future efforts to reduce harm and support thriving for GM individuals across the life span. Further, future work that explores the risk factors (e.g. race, education level, etc.) that play an important role in shaping the magnitude of the mental health disparity experienced by GMs, as well as how this magnitude varies across the life course, will be fruitful in further understanding the underlying patterns documented in the present study.

## Limitations

This study is subject to several limitations. Our measure of mental health was self-reported and did not capture specific symptoms or diagnoses (e.g., major depressive disorder; generalized anxiety disorder). Future work is needed to explore whether similar age patterns hold when using clinical measures. Additionally, the gender identity question is from an optional BRFSS module – to the extent that the 43 states that included this module may systematically differ regarding political and social climate toward gender minority individuals, our results may not fully be nationally representative. The BRFSS also does not survey people who are unhoused, are incarcerated, or reside in group-living quarters. Given that BRFSS is a cross-sectional survey, we cannot distinguish whether the observed differences are due to developmental age differences versus generational differences. Finally, sample size limited our ability to examine how disparities vary across other demographic factors (e.g., race, sexual orientation); future work should explore how intersectional identities impact the gender identity–based mental health age gradient disparities.

## Public health implications

Our findings demonstrate sizable GM inequities in mental health, though promising evidence of the size of these disparities reducing over the life course. Mental health and primary care providers must be prepared to address the unique psychosocial needs of GM adults, especially during emerging adulthood (ages 18–32) [9]. In order to effectively mitigate mental health disparities experienced by gender minority adults of all ages, future work is needed to understand factors that particularly drive poor mental health outcomes at various ages across the life course.

## Electronic supplementary material

Below is the link to the electronic supplementary material.

Supplementary Material 1

## Data Availability

The data are from the Behavioral Risk Factor Surveillance System. The data are available from https://www.cdc.gov/brfss/annual_data/annual_data.html. This is clearly stated within the manuscript.
