# Supplementary material for: The mental health age gradient by gender identity
Source: Soc Psychiatry Psychiatr Epidemiol. 2025 Apr 5;60(9):2271–3. doi: 10.1007/s00127-025-02895-3 (PMC12378824; doi:10.1007/s00127-025-02895-3)
Supplement: Supplementary file 1 — Supplementary Material 1 [file 127_2025_2895_MOESM1_ESM.docx]

| **Supplementary Table 1. Outcomes and Sociodemographic Characteristics by Gender Identity** | | |
| --- | --- | --- |
|  | **Cisgender** | **GM** |
| **Unweighted Sample Size** | N= 1,372,402 | N=6,498 |
| **Outcomes** | | |
| Bad Mental Health Days | 3.94 | 9.02* |
| **Demographics** | | |
| Sex |  |  |
| Female | 0.54 | 0.48* |
| Male | 0.45 | 0.51* |
| Missing | 0.0004 | 0.01* |
| Age | 44.51 | 55.31* |
| Race |  |  |
| White | 0.81 | 0.73* |
| Black | 0.08 | 0.10* |
| Asian | 0.04 | 0.06* |
| American Indian/ Alaskan Native | 0.02 | 0.03* |
| Other | 0.02 | 0.04* |
| Missing | 0.03 | 0.03* |
| Ethnicity |  |  |
| Hispanic | 0.08 | 0.13* |
| Missing | 0.01 | 0.01* |
| Education |  |  |
| No High School | 0.06 | 0.13* |
| High School Diploma | 0.26 | 0.31* |
| Some college | 0.28 | 0.27* |
| BA | 0.40 | 0.28* |
| Missing | 0.003 | 0.00 |
| LGB Status |  |  |
| Non-LGB | 0.92 | 0.45* |
| LGB | 0.05 | 0.52* |
| Missing | 0.02 | 0.04* |
| Married | 0.52 | 0.31* |
| Parenthood | 0.27 | 0.25* |
| Notes: Data come from the 2017-2022 BRFSS. Statistical significance reported from t-tests: * if p<0.05. | | |

| **Supplementary Table 2: Gender Minority Mental Health Disparity by Age Bin** | | | | | |
| --- | --- | --- | --- | --- | --- |
|  | **Unadjusted Disparity** | | | **Sample Size** | |
| **Age** | **Cisgender** | **Gender Minority** | **Difference**  **(and 95% CI)** | **Cisgender** | **Gender Minority** |
| 18-23 | 6.31 | 14.50 | 8.19  [7.15, 9.23] | 53,617 | 1,041 |
| 23-27 | 5.88 | 14.42 | 8.54  [7.18, 9.90] | 59,996 | 807 |
| 28-32 | 5.22 | 12.08 | 6.85  [5.02, 8.69] | 70,858 | 606 |
| 33-37 | 4.90 | 9.30 | 4.40  [2.48, 6.32] | 80,349 | 467 |
| 38-42 | 4.50 | 6.69 | 2.18  [0.37, 3.99] | 88,594 | 386 |
| 43-47 | 4.28 | 7.25 | 2.97  [1.07, 4.87] | 88,230 | 340 |
| 48-52 | 4.20 | 7.84 | 3.64  [1.65, 5.63] | 109,836 | 398 |
| 53-57 | 4.23 | 7.04 | 2.82  [-0.30, 5.94] | 122,556 | 426 |
| 58-62 | 3.97 | 4.41 | 0.44  [-1.88, 2.76] | 142,768 | 454 |
| 63-67 | 3.20 | 5.05 | 1.85  [0.27, 3.42] | 154,580 | 482 |
| 68-72 | 2.76 | 4.17 | 1.41  [-0.10, 2.92] | 146,235 | 423 |
| 73+ | 2.35 | 4.05 | 1.70  [0.13, 3.28] | 254,783 | 668 |
| Notes: CI = confidence interval. All statistics use BRFSS survey weights. | | | | | |

| **Supplementary Table 3: Gender Minority Mental Health Disparity by Age Bin** | | | | | | | | | | | | |
| --- | --- | --- | --- | --- | --- | --- | --- | --- | --- | --- | --- | --- |
| **Variable** | **18-23** | **23-27** | **28-32** | **33-37** | **38-42** | **43-47** | **48-52** | **53-57** | **58-62** | **63-67** | **68-72** | **73+** |
| GM | 4.22  [3.24, 5.19] | 4.42  [3.27, 5.57] | 3.83  [2.66, 5.00] | 2.42  [0.58, 4.27] | -0.05  [-2.16, 2.07] | 1.16  [-0.20, 2.51] | 2.69  [1.42, 3.97] | 2.13  [-1.33, 5.59] | -0.37  [-3.08, 2.35] | 1.39  [-0.21, 3.00] | 1.11  [-0.77, 2.99] | 1.44  [-0.24, 3.12] |
| Sex = Female | 2.21  [1.94, 2.48] | 1.76  [1.39, 2.13] | 1.63  [1.29, 1.97] | 1.48  [1.08, 1.87] | 1.53  [1.23, 1.83] | 1.66  [1.41, 1.92] | 1.73  [1.53, 1.93] | 1.79  [1.55, 2.04] | 1.67  [1.52, 1.81] | 1.14  [0.93, 1.35] | 0.75  [0.58, 0.93] | 0.42  [0.25, 0.59] |
| Sex = Missing | 6.70  [0.06, 13.33] | 4.73  [-5.32, 14.79] | 5.48  [-3.30, 14.26] | -0.40  [-4.08, 3.27] | 0.57  [-2.24, 3.39] | 4.14  [-1.05, 9.33] | 1.55  [-0.56, 3.66] | -0.92  [-3.75, 1.90] | 2.18  [-5.03, 9.39] | -1.11  [-1.84,  -0.39] | 1.54  [-0.14, 3.22] | 1.86  [-2.25,  5.97] |
| Age | 0.20  [0.10, 0.28] | -0.039  [-0.12, 0.05] | -0.01  [-0.11, 0.09] | -0.12  [-0.19, -0.04] | -0.04  [-0.10, 0.25] | -0.38  [-0.12, 0.05] | -0.13  [-0.24,  -0.23] | -0.03  [-0.09, 0.02] | -0.07  [-0.16, 0.01] | -0.11  [-0.18,  -0.05] | -0.06  [-0.11,  -0.02] | -0.12  [-0.15,  -0.08] |
| Race = White | 1.58  [1.19, 1.97] | 1.59  [1.20, 1.90] | 1.85  [1.51, 2.19] | 1.74  [1.22, 2.26] | 1.55  [1.06, 2.04] | 1.58  [0.98, 2.18] | 1.17  [0.70, 1.65] | 0.69  [0.14, 1.24] | 1.11  [0.41, 1.81] | 0.30  [-0.24, 0.85] | 0.57  [0.09, 1.06] | 0.32  [-0.26, 0.91] |
| Race = Black | 0.66  [-0.12, 1.43] | 0.28  [-0.29, 0.84] | 0.93  [0.39, 1.46] | 0.76  [-0.09, 1.61] | 0.67  [0.04, 1.30] | 1.09  [0.47,  1.72] | 0.68  [0.02, 1.38] | 0.22  [-0.34, 0.79] | 0.64  [-0.01,  1.29] | -0.34  [-1.03, 0.39] | 0.14  [-0.38, 0.66] | 0.35  [-0.24, 0.95] |
| Race = AIAN | 2.54  [0.67, 4.40] | 1.84  [0.77, 2.91] | 2.35  [1.64, 3.06] | 2.85  [2.21, 3.49] | 2.18  [1.11, 3.25] | 2.42  [1.75, 3.10] | 1.88  [0.93, 2.83] | 1.19  [0.41, 1.97] | 2.90  [1.50, 4.31] | 1.39  [0.63, 2.16] | 2.92  [0.85, 4.99] | 0.84  [0.12, 1.57] |
| Race = Other | 1.25  [0.32, 2.18] | 1.35  [0.81, 1.90] | 1.16  [0.48, 1.83] | 1.49  [0.65, 2.33] | 1.12  [0.31, 1.93] | 0.98  [0.01, 1.96] | 0.46  [-0.12, 1.05] | 0.16  [-0.81, 1.13] | 1.86  [1.12, 2.61] | 0.51  [-0.28, 1.29] | 0.73  [-0.18, 1.65] | 0.80  [-0.37, 1.97] |
| Race = Missing | 1.23  -0.01, 2.48] | 0.32  [-0.58, 1.21] | 0.44  [-0.10, 0.99] | 0.57  [-0.78, 1.21] | 0.78  [-0.16, 1.71] | 0.89  [-0.77, 1.85] | 0.21  [-0.61, 1.02] | 0.21  [-0.76, 1.19] | 1.24  [0.69, 1.80] | 0.36  [-0.60, 1.32] | 0.42  [-0.18, 1.02] | 1.60  [0.13, 3.06] |
| Ethnicity = Hispanic | -0.82  [-1.21,  -0.043] | -1.55  [-1.94,  -1.17] | -1.13  [-1.50,  -0.77] | -1.49  [-1.74,  -1.24] | -1.74  [-1.98,  - 1.51] | -1.55  [-1.92,  -1.19] | -0.98  [-1.49,  -0.47] | -1.42  [-1.92,  -0.92] | -0.94  [-1.42, -0.46] | -0.27  [-0.58, 0.03] | -0.28  [-0.67, 0.10] | -0.11  [-0,56, 0.34] |
| Ethnicity = Missing | -1.27  [-2.62, 0.07] | 0.33  [-0.86, 1.52] | 0.06  [-1.08, 1.20] | 0.88  [-0.28, 2.05] | 0.33  [-0.44, 1.11] | 0.27  [-0.92, 1.46] | 0.67  [-0.62, 1.97] | 0.53  [-0.63, 1.68] | 0.50  [-0.98, 1.98] | 0.89  [-0.18, 1.97] | 0.54  [-0.47, 1.56] | 0.60  [-0.18, 1.38] |
| Education = No High School | 2.30  [0.38, 4.22] | 1.81  [-0.49, 4.11] | 1.85  [0.49, 3.22] | 2.78  [1.73, 3.83] | 1.59  [-0.17, 3.31] | 1.78  [0.17, 3.39] | 3.43  [1.60, 5.26] | 3.65  [2.07, 5.23] | 0.39  [-3.02, 3.81] | 2.09  [1.04, 3.14] | 0.56  [-0.96, 2.09] | 0.48  [-0.84, 1.81] |
| Education = High School | 1.92  [0.01, 3.83] | 1.90  [-0.21, 4.01] | 1.80  [0.54, 3.07] | 2.29  [1.30, 3.27] | 0.75  [-1.08, 2.58] | 0.44  [-1.18, 2.07] | 1.72  [0.26, 3.17] | 1.51  [0.01, 3.01] | -1.15  [-4.21, 1.90] | 0.84  [-0.23, 1.92] | 0.08  [-1.59,  1.76] | -0.46  [-1.77, 0.85] |
| Education = Some College | 1.79  [-0.10, 3.68] | 1.79  [-0.51, 4.09] | 2.10  [0.85, 3.35] | 2.98  [2.18, 3.78] | 0.78  [-0.99, 2.55] | 0.59  [-1.01, 2.18] | 1.36  [-0.07, 2.80] | 1.45  [-0.01, 2.91] | -1.30  [-4.19, 1.59] | 0.78  [-0.30, 1.86] | -0.03  [-1.66, 1.59] | -0.41  [-1.72, 0.89] |
| Education = BA or higher | 0.46  [-1.54, 2.47] | 0.36  [-1.91, 2.64] | 0.53  [-0.77, 1.83] | 1.49  [0.61, 2.37] | -0.47  [-2.28, 1.34] | -0.79  [-2.35, 0.76] | 0.28  [-1.17, 1.72] | -0.02  [-1.52, 1.48] | -2.53  [-5.62, 0.56] | -0.26  [-1.31, 0.86] | -0.93  [-2.51, 0.64] | -0.97  [-2.29, 0.36] |
| Married | -0.99  [-1.66,  -0.31] | -1.80  [-2.14,  -1.47] | -1.56  [-1.96,  -1.17] | -1.89  [-2.12,  -1.65] | -2.13  [-2.45, 1.82] | -1.91  [-2.30,  -1.52] | -2.32  [-2.46,  -2.18] | -2.55  [-2.88,  -2.21] | -2.43  [-2.68,  -2.19] | -1.70  [-1.93,  -1.47] | -1.09  [-1.27,  -0.90] | -0.46  [-0.56,  -0.36] |
| Parent | -0.36  [-0.64,  -0.07] | -0.40  [-0.69,  -0.11] | -0.57  [-0.87,  -0.27] | -0.62  [-0.85,  -0.38] | -0.77  [-1.03,  -0.51] | -0.64  [-1.05,  -0.24] | -0.11  [-0.26, 0.03] | 0.11  [-0.05, 0.28] | 0.32  [-0.15,  0.79] | 0.26  [-0.09, 0.62] | 0.62  [0.11, 1.14] | 0.57  [0.12, 1.02] |
| Sexual Identity = LGB | 4.92  [4.35, 5.50] | 4.95  [4.47, 5.44] | 3.52  [3.00, 4.04] | 3.26  [2.85, 3.67] | 3.08  [2.63, 3.43] | 2.09  1.64, 2.54] | 2.07  [1.53, 2.62] | 1.14  [0.93, 1.79] | 1.18  [0.67, 1.69] | 0.93  [0.44, 1.43] | 1.14  [0.68, 1.61] | 0.66  [0.42, 0.89] |
| Sexual Identity = Missing | -0.32  [-1.34, 0.69] | -1.81  [-2.26,  -1.37] | -0.55  [-1.58, 0.48] | -0.54  [-1.46, 0.39] | -1.62  [-2.07, 1.18] | -1.42  [-2.13,  -0.71] | -1.44  [-2.12,  -0.77] | -0.45  [-1.40, 0.51] | -0.70  [-1.34,  -0.55] | -0.92  [-1.32,  -0.52] | -0.24  [-0.71, 0.22] | -0.03  [-0.18, 0.12] |
| Notes: CI = confidence interval. All statistics use BRFSS survey weights. Models were estimated using ordinary least squares and adjust for the variables listed as well as state and year. Standard errors are clusters at the state level. | | | | | | | | | | | | |
